# Long-Term Remission Achieved by Ponatinib and Donor Lymphocytes Infusion in a Ph+ Acute Lymphoblastic Leukemia Patient in Molecular Relapse After Allogenic Stem Cell Transplant and Dasatinib: A Case Report

**DOI:** 10.3389/fonc.2020.00967

**Published:** 2020-06-18

**Authors:** Cirino Botta, Nadia Caruso, Sabrina Bossio, Francesca Storino, Giuseppe Console, Massimo Martino, Francesco Mendicino, Eugenio Lucia, Rosellina Morelli, Pierpaolo Correale, Fortunato Morabito, Massimo Gentile, Ernesto Vigna

**Affiliations:** ^1^Hematology Unit, Hematology and Oncology Department, “Annunziata” Hospital of Cosenza, Cosenza, Italy; ^2^Biotechnology Research Unit, Hematology and Oncology Department, “Annunziata” Hospital of Cosenza, Cosenza, Italy; ^3^Stem Cell Transplant Program, Clinical Section, Department of Hemato-Oncology and Radiotherapy, “Grande Ospedale Metropolitano Bianchi-Melacrino-Morelli”, Reggio Calabria, Italy; ^4^Medicine Department, “Annunziata” Hospital of Cosenza, Cosenza, Italy; ^5^Medical Oncology Unit, Department of Hemato-Oncology and Radiotherapy, “Grande Ospedale Metropolitano Bianchi-Melacrino-Morelli”, Reggio Calabria, Italy; ^6^Hematology Department and Bone Marrow Transplant Unit, Cancer Care Center, Augusta Victoria Hospital, East Jerusalem, Israel

**Keywords:** T lymphocytes, donor lymphocyte infusion (DLI), ponatinib, bone marrow microenviroment, acute lymhoblastic leukemia

## Abstract

Currently, the prognosis of Ph+ acute lymphoblastic leukemia (Ph+ ALL) patients relapsing after an allogenic hematopoietic stem cell transplantation (allo-SCT) remains poor, with few therapeutic options available. Here we present the case of a 32 years old patient with dasatinib-resistant post-transplant molecular relapse of ALL, who received, as second-line therapy, the combination of ponatinib and donor lymphocyte infusion (DLI). The therapy was safe and the patient achieved a sustained minimal residual disease negative disease, still ongoing after 22 months, which was accompanied by several changes in the immune populations distribution within the bone marrow (i.e., the increase in the CD8/CD4 lymphocytes ratio). Our report provides evidence of the efficacy of the third generation TKI inhibitor ponatinib in combination with DLI as second line therapy for Ph+ ALL relapsing after an allo-SCT.

## Background

B cell Acute lymphoblastic leukemia (B-ALL) is a hematologic malignancy arising from B-cell progenitors that accounts for 20% of adult leukemias (1). Among ALL adult patients, approximately 25% presents an acquired chromosomal abnormality, the Philadelphia chromosome (Ph), resulting from a balanced translocation between chromosome 9 and 22 (2, 3), which leads to the formation of the hybrid BCR-ABL transcript. The occurrence of Ph chromosome increases with age and is associated with a worse prognosis (4). From a clinical point of view, Ph+ ALL usually presents with a higher white blood cell count (as compare to Ph- ALL) and with a 5% risk of central nervous system involvement at diagnosis (3). The 5 year survival rate range from about 40% for people aged 25–64 years to <15% for patients over 65 years (1). Tyrosine kinase inhibitors (TKIs) directed to BCR-ABL fusion protein (imatinib, dasatinib, ponatinib) represent the backbone of current therapy, achieving, when used alone or in combinatory schedules including chemotherapy, a hematological remission rate of about 90%. Allogenic hematopoietic stem cell transplantation (allo-SCT) still remains the only potentially curative treatment for patients in first remission; it should be noted, however, that it relies on patient “fitness” as well as on the availability of suitable donors, and is associated with a significant risk of morbidity and mortality (1). Despite the advances made since TKIs introduction, disease relapse remains the main cause of treatment failure. Indeed, relapsed or refractory ALL patients have dismal outcome with a median overall survival (OS) shorter than 1 year and a 3 year OS of <25% (5). To date, various therapeutic strategies are available for these patients including immunotherapy [donor lymphocyte infusions (DLI), blinatumomab, inotuzumab ozogamicin, or chimeric antigen receptor T cells (CAR-T)] as well as conventional cytarabine-based chemotherapy (CHT) regimens, a second allo-SCT, participation in a clinical trial or supportive care (5). DLI could induce remission in some patients by restoring the graft-vs.-tumor response; the anti-CD22 antibody-drug-conjugated (ADC) inotuzumab and the bispecific T cell engager (BiTE) blinatumomab demonstrated improved outcome as compared to standard salvage chemotherapy; the CD19-directed CAR-T tisagenlecleucel achieved 83% remission rate in pluri-treated young and adult patients with precursor B-ALL (5). Any of the previously described new drugs brings a series of new adverse events spanning from graft-vs.-host disease (GVHD) (with DLI) to cytokine release syndrome (CRS) and neurotoxicity (with blinatumomab and CAR-T) that could be life-threatening and should be carefully took into account during the decision-making phase.

In this case report, we describe a young woman presenting with molecular relapsed Ph+ B-cell ALL initially treated with CHT+TKI induction regimen followed by a matched related donor allo-SCT and maintenance treatment with TKI until relapse occurred. The patient achieved then a long term complete molecular and cytogenetic remission following Ponatinib+DLI with minimal toxicity.

## Case Presentation

**Figure 1:**
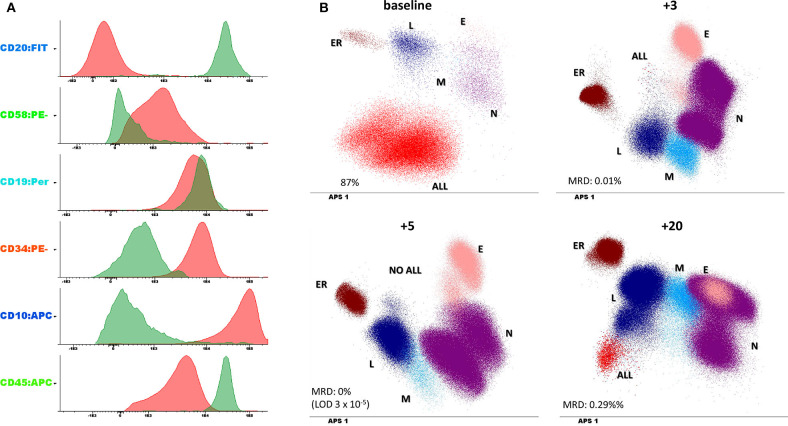
**(A)** Leukemic phenotype at baseline. The histograms report both the leukemic clone (in red) and the normal B cell compartment (in green) to underscore the expression of aberrant markers. (**B)** minimal residual disease (MRD) evaluation performed at 4 different timepoints (the number on the top of each panel represents the number of months from diagnosis) and compared with baseline phenotype. Dotplots are represented with a dimensionality reduction approach (principal component analysis or automatic population separator (APS) in infinicyt software, Cytognos) which allow the identification of the different immune population within the bone marrow (L, lymphocytes; ER, erythroblasts; N, granulocytes; M, monocytes; E, eosinophils; ALL, leukemic clone).

**Figure 2:**
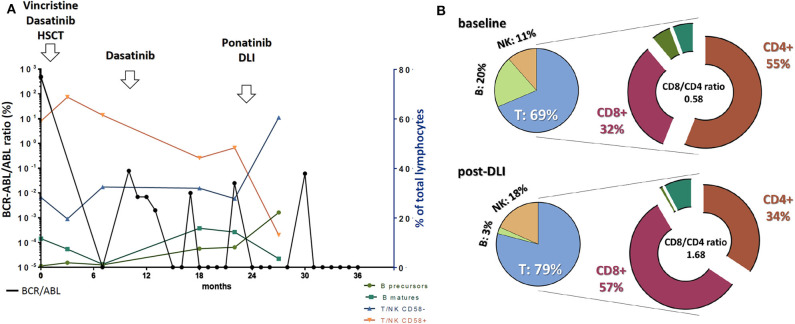
**(A)** Trends in BCR-ABL transcript levels in peripheral blood coupled with different normal immune populations distribution within the bone marrow along disease evolution and in response to different treatments. BCR-ABL/ABL ratio values are reported on the left vertical axis while cell population abundance refers to the right axis. (**B)** T, NK, and B cell representation within the whole lymphocyte group at baseline and after ponatinib + donor lymphocytes infusion (DLI). Notably, within the T cell compartment we observed an inversion in the CD8/CD4 ratio, favoring the CD8 compartment after DLI treatment.

A 32 year-old woman without significant comorbidities (with the exception of favism) presented to the emergency room with asthenia in March 2016 (all information reported in Table 1). Routine blood count showed lymphocytosis (white blood cell count: 40,000/mmc; platelets: 87,000/mmc; hemoglobin: 8.7 g/dl) with 80% lymphoid blasts in a peripheral blood smear. The subsequent bone marrow (BM) aspiration confirmed the diagnosis, showing the presence of aberrant cells with a B precursor immunophenotype (CD19+CD20–CD10brightCD45dimCD34+CD58+CD5–CD38low) (Figure 1A) and expression of the BCR-ABL1 p210 fusion gene. Flow cytometric analysis of cerebrospinal fluid revealed no central nervous system involvement. On May 5, 2016, the patient started an induction treatment with Vincristine 2 mg weekly (4 administrations), methylprednisolone (60 mg/m^2^/day for 28 days) and dasatinib 140 mg daily (early switch from imatinib due to intolerance after the first 7 days of treatment), associated with the intrathecal administration of cytarabine and methylprednisolone as a prophylaxis, which resulted in a rapid hematological complete response and BM negative minimal residual disease (MRD) (10^−4.2^ in PCR, 10^−4.5^ in FCM) after 5 months (Figure 1B). On October 2016, the patient underwent an allo-SCT (4.6 × 10^6^ CD34+ cells /Kg) from a HLA-matched relative (brother), after a myeloablative conditioning regimen that included busulfan and cyclophosphamide (BuCy2) plus antithymoglobulin (ATG). As graft-vs.-host disease (GVHD) prophylaxis the patient received cyclosporine and methotrexate. Grade 2 fever and grade 1 mucositis and diarrhea were the main acute toxicities observed during hospitalization. On February 2017, for the reappearance of BCR-ABL1 transcript (BCR-ABL/ABL ratio in peripheral blood: 0.079%), the patient stopped (progressively) cyclosporine treatment and re-started dasatinib. On day 200 after transplant the detected chimerism was 100%. MRD negativity was then maintained until February 2018, when the patient experienced a molecular relapse while staying in morphological CR (BCR-ABL/ABL ratio in peripheral blood: 0.025%). Of note, the patient was negative for the T315I mutation (conventional Sanger sequencing). The therapeutic decision, at this point, was to change the TKI, by switching to ponatinib (45 mg once a day), and to begin DLI (5 consecutive infusions until September 2018). The patient quickly achieved a complete molecular response (CMR) (MR4.5) but experienced signs of GVHD with the development of progressively expanding dyskeratosis and dry eye (confirmed with Schirmer test) for which begun a treatment with steroids and cyclosporin. Central nervous system evaluation through MNR and cerebrospinal fluid analysis reveal no signs of leukemic involvement. Interestingly, this clinical picture was accompanied by a substantial increase in normal B cell progenitors, an inversion between CD58+ and CD58– T/NK lymphocytes distribution (Figure 2A) and by an inversion of the CD8/CD4 ratio (Figure 2B) within the BM. At the last evaluation, done on December 2019, the patient is continuing therapy with ponatinib 45 mg once a day with reasonable tolerance and still maintains a MR4.5 (response maintained for 22+ months).

**Table 1:** Patient's main characteristics at baseline and along the treatment.

**Case presentation**	
Classification	B-precursor ALL Ph+
WBC (μl)/Hb (g/dl)/platelets (μl)	40,000/8.7/87,000
Blasts	80%
Blasts phenotypes	CD19+, CD20–, CD10bright, CD45dim, CD34+, CD58+, CD5–, CD38low
BCR-ABL isoform	P210
Induction regimen	Vincristine + Dasatinib
CD34+ cell dose during transplant	4.6 × 10^6^/kg
GVHD prophylaxis	Cyclosporin, MTX
Time from transplantation to relapse (months)	16
Treatment after relapse	DLI + ponatinib
Current status	Alive in molecular remission
Time from diagnosis (months)	45

## Discussion

The management of allo-SCT relapsed Ph+ ALL patients represents a great challenge due to few active therapeutic regimens that rarely are successful, rendering the prognosis very poor (6). Indeed, contrary to what happens in chronic myelogenous leukemia (CML), TKIs are less effective in B-ALL scenario due to the rapid onset of drug resistance (7). Additionally, molecular detection of relapse (such as in the case of MRD reappearance) could help to identify patients who could potentially benefit from an early salvage therapy. This point is currently under active investigation, with increasing data suggesting that after an allo-SCT, the loss of MRD negativity, as measured by reappearance of BCR-ABL transcript within the bone marrow, precedes hematologic relapse, therefore justifying an early initiation of the treatment (8, 9). In this regard, in both Ph– and Ph+ ALL and detectable MRD, therapy with blinatumomab (alone or in combination with TKI, respectively) has shown encouraging results, and larger clinical trials are ongoing (10, 11). However, as previously discussed, Ph+ ALL patients relapsing after an allo-SCT, currently undergo a second line therapy which could include (1) an alternative TKI (alone or in combination with chemotherapy or steroids); (2) blinatumumab; or (3) the anti-CD22 (if present on leukemic clone) inotuzumab ozogamicin, being the latter two indicated for TKIs intolerant or refractory patients only. Tisagenlecleucel represents a further therapeutic option for Ph+ ALL patients up to 25 years after at least 2 relapses or failure of 2 TKIs. Additionally, a second allogenic allo-SCT or DLI could be considered for relapsed patients, even if data are currently unclear. Here we reported a case of a post-transplant molecular relapsed ALL patient treated with the combo ponatinib-DLI, who achieved a sustained long-term response with MRD negativity (+22 months). TKI inhibitors are known to be unable to eradicate leukemic clones, mainly due to the fact that stem leukemic clones are often Ph- or BCR-ABL independent, thus being not targetable with current drugs (12). On the other hands, the infusion of DLI from an allogeneic donor could induce deep remissions but at the cost of significant toxicity (such as GVHD) (13, 14). On these bases, the combination of a TKI inhibitor (to deeply reduce tumor burden) with DLI (to try to eradicate residual clonal cells) could represent a reasonable option to produce long lasting remissions. Previous case reports explored the feasibility of the combination of both 1st and 2nd generation TKI with DLI (5, 15–17), with alternate results (Table 2). Specifically, imatinib + DLI used at first relapse after allo-SCT (but not at late relapse) was followed by CMR achievement lasting over 24 months with negligible signs of GVHD (15). Furthermore, in imatinib resistant patients, the combination of 2nd generation TKI Dasatinib or Nilotinib + DLI achieved MRD negativity and CMR, respectively. Interestingly, Nilotinib + DLI used at 4th relapse, surprisingly induced a CMR lasting over 10 months, with a manageable grade 1 cutaneous and hepatic GVHD (16). Along this line, in our scenario (Dasatinib-resistant patient), the combination of the 3rd generation TKI inhibitor ponatinib with DLI represented an attractive therapeutic opportunity.

**Table 2:** Comparison of published case reports on the combination of DLI+TKI in T-ALL.

	Yoshimitsu et al. (15)	Tiribelli et al. (16)	Tachibana et al. (17)	Maharaj et al. (5)
WBC (μl)/Hb (g/dl)/platelets (μl) at diagnosis	11,700/10.2/28,000	NA	40,800/NA/NA	67,000/12.3/49,000
BM Blasts	98.6%	NA	97%	NA
Blasts phenotypes	CD10^+^, CD13^+^, CD19^+^, CD34^+^,CD33^+^	NA	CD10^+^, CD13^+^, CD19^+^, CD34^+^, HLA-DR^+^	NA
BCR-ABL isoform	p190	p190	NA	p190
SNC involvement	NA	Meningeal and ocular	NA	NA
1th Induction regimen	ALL202 (prednisolone, CPA, daunorubicin, VCR) + imatinib + intrathecal CHT (methotrexate, ARA-C and DEX)	VCR, daunorubicin, l-asparaginase and prednisone + intrathecal CHT (methotrexate, ARA-C and DEX)	Prednisolone, DXR, vindesine and CPA + imatinib	UKALL14 (PEG-asparaginase, daunorubicin, VCR, CPA, ARA-C, mercapopurine, DEX + + intrathecal CHT (methotrexate)
1th consolidation regimen	HD MTX and HD ARA-C	HAM protocol (HD ARA-C and mitoxantrone) + CNS radiotherapy	MTX, ARA-C, and methylprednisolone	CPA + total body irradiation
Response to 1th treatment	CCR	CCR	CMR	CMR
CD34+ cell dose during transplant	5.0 × 10^6^/kg	3.0 × 10^6^/kg	NA	NA
Time from allo-SCT to first relapse (months)	4.5	5.5	3.0	6
Treatment after relapse	Imatinib ± DLI	Imatinib BFM protocol (VCR, ifosfamide, MTX, ARA-C, and teniposide) DLI + VCR + prednisone Nilotinib ± DLI	imatinib, CPA, DXR, VCR and prednisolone Imatinib ± DLI imatinib, CPA, DXR, VCR, and prednisolone Dasatinib	Dasatinib ± DLI Dasatinib + IL-2
Best response to TKI + DLI	CMR	CMR	CCR maintenance	CCR
Signs of GVHD	ANA+	grade I skin and liver GVHD	None	NA
Last reported status	CMR at 24 months after Imatinib + DLI start	CMR at 10 months after Nilotinib + DLI start	Relapse at 8 months after Imatinib + DLI start	Relapse at 6 months after Dasatinib + DLI start

*HD, high doses; MTX, methotrexate; ARA-C, cytarabine; VCR, vincristine; CPA, cyclophosophamide; DEX, dexamethasone; DXR, doxorubicin; DLI, donor lymphocytes infusion; CCR, complete cytogenetic remission, CMR, complete molecular remission; allo-SCT, allogenic stem cell transplant; NA, not available*.

Accordingly, we quickly achieved a molecular response with no signs of acute GVHD other than a manageable cutaneous dyskeratosis, and the patients received 5 consecutive DLI injections, continuing to maintain a complete molecular response. Interestingly, the treatment combination produced several immune changes within the BM, that are in line with the reported immunomodulatory activity of this class of drugs (18, 19). We indeed observed a “normalization” in bone marrow composition (including a repopulation in precursor and naïve b and T cells) coupled with an unbalance in the CD8/CD4 ratio in favor of the cytotoxic CD8 T cell population, known to be essential for anticancer response (20). As a result, the patient achieved a quick and sustained complete molecular response with the development along the time, of manageable dyskeratosis and is still in complete molecular remission. Summarizing, we believe ponatinib in combination with DLI a very active regimen in ALL patients after the failure of a first line TKI and allo-SCT. Clinical trials are eagerly awaited to confirm its therapeutic potential in this setting.

## Ethics Statement

Written informed consent was obtained from the individual for the publication of any potentially identifiable images or data included in this article.

## Author Contributions

CB composed the manuscript and performed literature review. NC, SB, and FS did the acquisition and analysis of laboratory data for the work. CB and EV critically revised and interpreted the data. EV, GC, MM, FMe, EL, RM, FMo, MG, and CB took care of the patient from the clinical point of view. CB and EV wrote the manuscript while MG and PC fully revised and improved it. All authors contributed to the article and approved the submitted version.

## Conflict of Interest

The authors declare that the research was conducted in the absence of any commercial or financial relationships that could be construed as a potential conflict of interest. The handling editor declared a past co-authorship with one of the authors MG.
